# Impact of Discharge Rounds on Patient Flow and Hospital Outcomes

**DOI:** 10.7759/cureus.92267

**Published:** 2025-09-14

**Authors:** George Bechir, Mishame Anja

**Affiliations:** 1 Hospital Medicine, Franciscan Health, Munster, USA; 2 Medicine, Black Lion Specialized Hospital, Addis Ababa, ETH

**Keywords:** acute care hospitals, discharge planning, discharge rounds, hospital efficiency, hospital length of stay, interdisciplinary communication, multidisciplinary rounds, patient flow, patient safety, virtual rounds

## Abstract

Discharge delays are a common and costly problem in hospitals, exposing patients to preventable risks while straining system capacity. Discharge rounds are structured daily meetings designed to identify barriers and coordinate care, and have emerged as a potential solution, yet implementation varies widely across institutions. This narrative review examined 38 studies published between 2010 and 2024 that evaluated discharge rounds in adult acute care hospitals. The evidence demonstrated that outcomes depend more on execution than on the concept itself. Hybrid formats that combine in-person and virtual participation improved attendance and preserved communication quality. Early morning rounds of limited duration facilitated timely discharges and aligned better with hospital operations. A lean but interdisciplinary core team of hospitalists, case managers, and nurses was consistently effective, with pharmacists and social workers adding value in select populations. Nursing participation models required tailoring. Bedside nurse involvement proved beneficial in high-acuity settings, while charge nurse representation was effective in more stable units. Geographic cohorting of physicians reduced inefficiency from multi-floor coverage, although handoffs raised continuity concerns. Across all models, the most successful rounds focused their discussion on discharge readiness, specific barriers, assigned responsibilities, and clear timelines rather than broad clinical debates. Programs that lacked structure or drifted from this focus often failed to achieve measurable benefits. In conclusion, discharge rounds can reduce length of stay and improve hospital efficiency, but only when carefully designed. Hospitals seeking to implement or refine discharge rounds should prioritize format, timing, team composition, and discussion focus while adapting to local context.

## Introduction and background

Walk into any hospital at 3 PM and you'll likely find the same scene: patients dressed and ready to go home, sitting on their beds, waiting. Maybe they are waiting for one last consultant note. Perhaps their ride cannot come until after work. Or possibly, and this happens more often than hospitals like to admit, no one quite knows what they are waiting for. This scenario plays out daily across American hospitals, contributing to what researchers call avoidable hospital days. These are not medically necessary days; they are coordination failures. And they carry both financial costs and clinical risks, exposing patients to hospital-acquired infections, deconditioning, and the psychological toll of feeling stuck [1,2].

Discharge rounds are structured, team-based discussions focused on identifying and removing barriers to timely discharge. When designed well, they can shorten hospital stays, reduce readmissions, and improve communication among providers and patients.

Discharge rounds are organized meetings held each day, where the care team gathers to identify barriers, coordinate plans, and push discharges forward. The idea is simple, but execution varies widely. Some hospitals conduct rounds at the bedside, others in conference rooms, and since the COVID-19 pandemic, many have gone virtual. The composition of the team, the timing of the meetings, and the duration also differ from one hospital to another.

These differences matter. Research has shown that discharge processes strongly influence how long patients remain in the hospital, and structured approaches can improve both efficiency and patient experience [3]. Reviews confirm that discharge rounds can reduce length of stay and improve outcomes, but results remain inconsistent and depend heavily on how rounds are designed and implemented [4,5].

This review focuses on the critical questions hospitals face when implementing discharge rounds. What format works best: in person, virtual, or hybrid? When should rounds occur, and how long should they last? Which disciplines must be present, and what role should nurses play? How should physicians manage patients scattered across multiple units? What should be discussed, and what should be left out? Finally, do discharge rounds free up more time for direct patient care, or do they risk becoming just another meeting?

By addressing these questions, this review aims to clarify which discharge round practices reliably improve patient flow and care quality and which risk becoming burdensome without measurable benefit.

## Review

Methodology

We conducted a narrative review of the literature examining discharge rounds and their impact on hospital length of stay. PubMed, Cumulative Index to Nursing and Allied Health Literature (CINAHL), and Google Scholar were searched for articles published between January 2010 and December 2024 using combinations of terms including "discharge rounds," "multidisciplinary rounds," "length of stay," "virtual rounds," "nurse participation," "physician workflow," and "geographic cohorting." Studies were included if they evaluated discharge or multidisciplinary rounds in adult acute care hospitals, reported outcomes related to length of stay or discharge efficiency, and described implementation details such as format, timing, or team composition. We excluded studies conducted in pediatric hospitals, psychiatric or rehabilitation facilities, as well as editorials, commentaries, and opinion pieces without original data.

The initial search yielded 342 articles. After removing duplicates and screening titles and abstracts, 89 full-text articles were reviewed. Following application of inclusion and exclusion criteria, 38 studies were selected for analysis, comprising 15 prospective observational studies, seven retrospective cohort studies, eight quality improvement reports, five randomized trials, and three systematic reviews.

Data extraction focused on round format (in-person, virtual, or hybrid), timing and duration, team composition, impact on length of stay, nursing participation models, physician coverage strategies for multiple floors, management of inter-unit patient transfers, and effects on physician-patient interaction time. Special attention was paid to how hospitals handled physician coverage when patients were scattered across units and whether care continuity was maintained when patients moved between floors.

Given the heterogeneity of interventions and outcomes, we performed a narrative synthesis organized around key implementation questions: optimal format, timing, team composition, nursing roles, multi-floor coverage strategies, and impact on care quality. We examined patterns across successful and unsuccessful implementations, noting contextual factors that might explain varying results.

Limitations

As this was a narrative review, no meta-analysis was performed. Heterogeneity in study designs, patient populations, and outcome measures limited direct comparability across studies. Because this was a narrative review, no formal risk of bias assessment was conducted; instead, study design and context were considered when weighing findings, with greater weight given to randomized trials and systematic reviews compared to observational or quality improvement reports.

Format of discharge rounds: in person, virtual, or hybrid

The traditional model of in-person discharge rounds dominated until 2020, when COVID-19 forced rapid adoption of virtual alternatives. Our review found studies directly comparing formats, with results that challenge conventional assumptions about the superiority of face-to-face communication.

In-person rounds showed consistent benefits in earlier studies. O'Leary et al. examined structured interdisciplinary bedside rounds across multiple units and found that bedside rounds reduced length of stay compared to conference room rounds [6]. The bedside format allowed real-time assessment of patient readiness and immediate clarification of ambiguous clinical findings. Nurses particularly valued bedside rounds, with most reporting improved understanding of discharge plans when rounds occurred at the bedside rather than in conference rooms [7].

The pandemic necessitated virtual rounds, providing a natural experiment. Virtual team rounding programs were rapidly implemented to manage COVID-19 surges and maintain care coordination when in-person gathering was restricted [8]. Length of stay remained stable while round duration decreased and specialist participation increased when consultants could join remotely rather than travel to specific units.

The most compelling evidence supports hybrid models combining virtual and in-person elements. Virtual communication embedded with bedside rounds demonstrated how hybrid approaches could maintain quality while improving efficiency [9]. These hybrid rounds, where the core team (attending physicians, residents, nurses, and case managers directly responsible for daily patient care and discharge planning) meets in person while specialists and consultants join virtually and actively participate in real-time discussion and decision-making, achieved better attendance rates and preserved clinical decision-making quality compared to either pure virtual or pure in-person formats. The hybrid model captured efficiency gains from virtual participation while preserving the communication benefits of in-person interaction for the core treating and discharge planning team.

Implementation details mattered. Successful virtual components required reliable technology, clear audio protocols, and screen-sharing capability for imaging review. Failed implementations often cited poor audio quality, lack of visual cues, and technology frustrations that prolonged rather than shortened rounds. To synthesize the evidence across formats, Table 1 provides a side-by-side comparison of in-person, virtual, and hybrid discharge rounds, while Table 2 summarizes the strengths, limitations, and key evidence of discharge round formats.

**Table 1:** Summary comparison of discharge round formats

Feature	In-Person Rounds	Virtual Rounds	Hybrid Rounds
Communication Quality	Highest: Allows for non-verbal cues and immediate clarification at the bedside.	Moderate: Risk of miscommunication due to technology failures or a lack of visual cues.	High: Preserves in-person dynamics for the core team while allowing clear remote input.
Efficiency and Duration	Least efficient: Can be prolonged; requires travel time for all participants.	Most efficient: Shorter duration and no travel time for participants.	Highly efficient: Eliminates travel for specialists and keeps core discussion focused.
Specialist Participation	Lowest: Difficult to coordinate physical attendance for brief patient discussions.	High: Enables specialists to join remotely for relevant cases without disrupting their workflow.	Highest: Offers the easiest and most effective way to integrate specialist input as needed.
Implementation Needs	Co-located physical space (conference room or bedside).	Reliable audio/video technology, strong Wi-Fi, and clear protocols for all participants.	Both a physical meeting space and reliable virtual conferencing technology.
Key Advantages	Best for complex, hands-on patient assessments and team bonding.	Maximum flexibility, safety during outbreaks, and operational efficiency.	Optimal balance of communication quality, efficiency, and expert participation.

**Table 2:** Strengths, limitations, and key evidence of discharge round formats

Format	Strengths	Limitations	Key Evidence
In-person	Real-time bedside assessment; stronger rapport with patients and nurses; direct communication	Time-intensive; harder for consultants to attend multiple units; requires travel	O’Leary et al. 2010 [6]; Gonzalo et al. 2014 [7]
Virtual	Increased consultant participation; reduced travel time; shorter duration	Risk of disengagement; technology issues; less effective for complex discussions	Becker et al. 2021 [8]; Bavare et al. 2021 [9]
Hybrid	Combines bedside presence with remote consultant input; maintains decision quality and efficiency	Requires reliable technology and clear protocols; more complex to organize	Bavare et al. 2021 [9]

Optimal timing and duration

Timing emerged as a critical factor affecting discharge efficiency, with studies reporting that earlier rounds were consistently associated with better discharge outcomes. Evidence strongly supported morning rounds, particularly those held before mid-morning, as they allowed discharge orders to be placed earlier in the day and facilitated completion of tasks such as pharmacy processing, patient education, and transportation during business hours.

Several studies highlighted the advantages of starting discharge rounds before 9 AM, typically involving the core bedside team (attending or hospitalist physician, case manager, and bedside or charge nurse, with residents included where applicable). Early timing ensured that night shift nurses were still available to provide overnight updates, morning laboratory results were accessible, and case managers could act on identified barriers during standard working hours. Hospitals that adopted this approach reported higher rates of before-noon discharges [10], an important operational benchmark linked to improved patient flow [11].

Duration also influenced outcomes. Studies found that rounds lasting fewer than 15 minutes often lacked sufficient time for meaningful problem solving, while rounds extending beyond 30 minutes showed diminishing returns [12]. After 30 minutes, discussions tended to become repetitive or drift away from discharge planning. Programs that enforced strict time management strategies, such as assigning a maximum of two minutes per patient, maintained efficiency while ensuring that key barriers were addressed. The basis for these findings came from observational studies across multiple hospitals, which consistently linked time discipline to better discharge planning outcomes [13].

The relationship between timing and hospital operations proved important. Hospitals that aligned discharge rounds with other workflows, such as morning physician rounds (to ensure clinical updates were immediately incorporated) or case management meetings (to accelerate post-acute planning and insurance coordination), achieved better integration and faster resolution of barriers. Conversely, rounds scheduled during peak clinical activity or shift changes often failed to achieve meaningful impact [14].

Team composition: who needs to be present?

The composition of discharge rounds varied significantly across studies, from lean three-person teams to comprehensive groups of twelve or more participants. The evidence suggests that core team composition matters more than total size, with certain roles being essential while others add value only in specific contexts.

The essential core team consistently included three roles: hospitalist or attending physician (a clinician directly responsible for inpatient medical management, not an administrator), case manager or discharge planner, and bedside nurse or charge nurse. Studies removing any of these three roles showed decreased effectiveness. The physician provides medical decision-making authority, the case manager coordinates post-acute services and insurance requirements, and the nurse offers real-time patient status updates. This triad formed the minimum effective team across all successful implementations [15].

Adding a clinical pharmacist as the fourth core member showed measurable benefits. Pharmacist participation reduced medication-related discharge delays and decreased readmissions related to medication errors [16]. Their role proved particularly valuable for patients on multiple medications or those requiring prior authorizations, anticoagulation management, or complex discharge prescriptions.

Social work involvement depended on the patient population. Units with high proportions of patients facing psychosocial barriers, homelessness, or substance use disorders benefited significantly from embedded social work participation. Social workers acted as key contacts for connecting patients to community resources, insurance services, and psychosocial support. However, having social workers on call rather than attending all rounds proved equally effective for units with lower psychosocial complexity, especially when supported by duty rosters or structured communication systems that ensured timely input on critical discharge decisions [17].

Physical and occupational therapy participation showed mixed results. Direct therapist involvement helped when mobility and functional status were primary discharge barriers. However, several successful programs used written therapy reports or brief verbal updates instead of therapist attendance, preserving therapy treatment time while maintaining communication [18].

Specialist involvement through virtual platforms emerged as an effective and widely recommended practice. Rather than requiring physical presence, having specialists available by video or phone for specific patients reduced delays while respecting time constraints. One program reported that optional specialist dial-in increased consultant input from 20% to 75% of relevant cases [19].

The inclusion of residents and students requires balance. Teaching programs that integrated education into discharge rounds without extending duration showed no negative impact. However, programs that allowed extensive teaching discussions during rounds saw increased length of stay and staff frustration. Successful programs designated specific teaching points or held separate teaching rounds [20].

Nursing participation: bedside care versus round attendance

The role of nurses in discharge rounds generated the most debate among the studied institutions, with fundamentally different philosophies about whether nurses should attend rounds or remain at the bedside. This question becomes even more complex given current nursing shortages and increased patient acuity.

Three distinct models emerged from the literature. The first requires bedside nurse attendance for their assigned patients. Proponents argue that nurses have the most current information about patient status, overnight events, and subtle changes that might affect discharge readiness. One study found that when bedside nurses attended rounds, discharge delays related to “patient not ready” decreased significantly [21]. Nurses could immediately clarify ambiguous situations, such as when the team assumed mobility independence but the nurse reported recent bedside assistance needs.

The second model uses charge nurses or designated discharge nurses to represent all patients on the unit. This approach preserves bedside nursing time while maintaining the nursing voice in rounds. A comparative study found this model achieved nearly the same reduction in length of stay as full bedside nurse participation while preserving additional direct care time for staff nurses [22]. Charge nurses developed expertise in discharge planning and could advocate for bedside nurse concerns without pulling multiple nurses away from patient care.

The third model excludes nurses from rounds entirely, relying on pre-round communication or electronic updates. While this maximizes bedside time, studies showed increased discharge delays due to miscommunication. Institutions that trialed this approach often reverted after discovering that “ready for discharge” determinations were frequently inaccurate without nurse input, leading to last-minute cancellations when issues such as uncontrolled pain or confusion were identified [23].

The optimal approach appears to depend on unit characteristics and patient acuity. Medical-surgical units with stable patients often succeed with charge nurse representation. Critical care and step-down units benefited most from bedside nurse participation, given the complexity and rapid changes in patient status. In these settings, charge nurses typically ensured overall coordination, while bedside nurses contributed real-time patient updates, discharge readiness assessments, and follow-through on discharge-related tasks as part of the core team. Surgical units frequently adopted brief nurse check-ins via phone or tablet rather than physical attendance.

Timing within the shift also played a role. Rounds held during shift changes consistently failed due to nursing unavailability, while those scheduled between 8 and 9 AM, after initial assessments but before peak medication administration, were the most successful [24].

Physician coverage across multiple floors: the geographic challenge

One of the most significant operational challenges identified was managing discharge rounds when physicians have patients scattered across multiple floors. The average hospitalist covers patients on more than three different units, and in tertiary centers, this number can be as high as six. This geographic dispersion creates a fundamental conflict between comprehensive discharge planning and efficient time utilization.

Traditional approaches requiring physicians to physically attend rounds on each floor proved unsustainable. Time-motion studies revealed that physicians often spent nearly an hour traveling between units each day, not including the time spent in rounds themselves. Observational research confirmed that scattered patient coverage led to wasted clinician time, fragmented communication, and delayed discharge decisions [25,26].

Virtual participation emerged as one solution. Physicians joined rounds remotely from a central location, cycling through different units via video connection. This eliminated travel time while maintaining physician input. However, pure virtual participation had drawbacks: nurses reported reduced physician engagement, and complex discussions were harder to manage through screens. To preserve patient-doctor trust, successful programs emphasized transparency, with bedside staff explaining the role of remote physicians and ensuring that patients and families could still ask questions or request in-person review when needed. In some cases, physicians still needed to visit patients separately at the bedside, offsetting the efficiency gains.

Geographic cohorting, where physicians are assigned to specific units rather than following patients across multiple locations, demonstrated the most consistent benefits. Hospitals that adopted geographic cohorting reported shorter lengths of stay, reduced readmission rates, and improved efficiency [13]. A cluster randomized trial confirmed that this model decreased paging burden and improved physician workflow, although continuity concerns emerged when patients were transferred between units [11]. Patients frequently expressed confusion and frustration when their admitting physician “disappeared” after a floor transfer. To address cross-consultation needs across specialties, successful programs supplemented geographic cohorting with virtual consults or scheduled in-person specialist visits, ensuring that patients continued to receive multidisciplinary input without compromising the efficiencies of cohorting.

Hybrid models attempted to balance efficiency with continuity. In the “primary plus geographic” approach, the admitting physician retained overall responsibility while a geographically based partner handled daily rounds and coordination. This model, implemented in accountable care unit redesigns, showed improvements in teamwork and discharge processes while still preserving a link to the primary physician [10].

Taken together, the evidence indicates that geographic cohorting is the most effective approach for discharge rounds, especially in improving efficiency and reducing delays. While hybrid models provide a compromise by maintaining continuity, the traditional scattered coverage model has consistently been associated with wasted clinician time and delayed discharges [25].

Content and focus: what should be discussed in discharge rounds

A critical factor distinguishing effective from ineffective discharge rounds was the content of discussions. Many failed programs became “mini medical rounds,” rehashing clinical details rather than focusing on discharge barriers. The evidence strongly supports a barrier-focused approach with specific talking points that drive action [4,6].

Successful programs followed a structured format for each patient, typically addressing five key questions in order. 

First: Is this patient medically ready for discharge today or tomorrow? This binary question prevented lengthy medical discussions. If not, the discussion moved to the next patient. If yes or possibly, the team proceeded to barrier identification. 

Second: What specific barriers prevent discharge? High-performing teams used standardized barrier categories: medical stability, functional status, equipment needs, medication access, transportation, placement or home services, and family readiness. In a multidisciplinary hospital-wide initiative, improving before-noon discharge rates from 14% to 24%, structured barrier tracking played a central role. One program’s analysis of 10,000 discharges found that 73% had barriers in just three categories (placement, equipment, and medication access), allowing targeted problem solving rather than general discussion [10].

Third: Who owns each barrier, and what’s the resolution timeline? Assigning specific ownership with deadlines transformed vague plans into actionable items. Instead of “working on placement,” the case manager would state, “Skilled nursing facility (SNF) bed confirmed for tomorrow, pending insurance authorization, which I’ll complete by noon.” This specificity created accountability and prevented barriers from lingering unaddressed.

Fourth: What’s the anticipated discharge date and time? Programs that assigned specific target dates and times (not just “probably tomorrow”) achieved significantly higher rates of before-noon discharges [10,11].

Fifth: What education or preparation does the patient need? Rather than assuming education would happen somehow, successful rounds explicitly assigned responsibility for patient teaching about medications, follow-up appointments, and warning signs.

Topics that should not dominate discharge rounds included detailed medical histories, teaching discussions about pathophysiology, social commentary, and extensive debate over clinical management. One successful program used a “parking lot” system, where important but non-discharge issues were noted for discussion after rounds.

The most effective programs also addressed contingency planning. Asking, “If the SNF bed falls through, what’s Plan B?” helped prevent last-minute scrambling when primary plans failed.

Impact on physician time and patient care quality

The most persistent concern about discharge rounds centers on a fundamental question: Does pulling physicians into 30- to 60-minute meetings improve or harm patient care? Critics argue that rounds remove physicians from the bedside during valuable morning hours, while supporters counter that better coordination actually increases meaningful patient interaction time. The evidence provides a nuanced answer.

Time-motion studies before and after discharge round implementation revealed surprising results. While physicians did spend an average of 45 minutes in discharge rounds, their total direct patient care time actually increased by nearly half an hour per shift. The explanation lies in efficiency gains: without structured rounds, physicians spent several hours each shift on fragmented coordination through phone calls, pages, and hallway conversations. Discharge rounds condensed these activities into a single focused session [14].

One multicenter study of hospitalists demonstrated that those participating in structured discharge rounds had 18% more bedside time compared with peers relying on traditional communication methods. Physicians described fewer interruptions and greater clarity in patient care tasks, reporting that “30 minutes in rounds saved hours of chasing down updates later” [24].

Quality metrics supported these efficiency gains. Patient satisfaction scores regarding physician communication increased after implementing patient-centered bedside rounds, with improvements in decision control, activation, and satisfaction [6].

However, negative effects emerged when rounds were poorly implemented. Programs where rounds exceeded an hour or lacked focus saw physician frustration increase and patient care time decrease. When discharge rounds devolved into unfocused clinical discussions rather than targeted planning, the intended efficiency benefits were lost [13].

The timing of rounds within the physician workflow also mattered. Programs that intentionally positioned rounds alongside early-day workflows reported better integration and fewer interruptions; structured rounding tools that standardized discussion order and timeboxing improved time allocation and communication efficiency [27].

## Conclusions

Discharge rounds represent a powerful yet underutilized tool for reducing hospital length of stay and improving care coordination. The evidence from this review demonstrates that when discharge rounds are implemented with structure and discipline, they not only shorten hospital stays but also enhance efficiency, increase meaningful physician-patient interaction, and improve patient satisfaction. Conversely, poorly executed rounds risk becoming another time-consuming meeting that detracts from care.

Several key lessons emerge. First, hybrid formats that combine in-person participation for core team members with virtual attendance for specialists offer the most effective balance of efficiency and communication. Second, early morning rounds limited to 20-30 minutes maximize the opportunity to complete discharge tasks within business hours while avoiding workflow fragmentation. Third, the essential core team should always include the hospitalist, case manager, and nurse, with pharmacy and social work involvement tailored to patient needs. Fourth, nursing participation models must adapt to unit context, balancing bedside availability with the need for discharge input. Finally, geographic cohorting consistently outperforms scattered physician coverage in supporting timely discharges, with hybrid models offering a compromise where continuity must be preserved.

Above all, the most effective discharge rounds maintain a laser focus on barriers to discharge, assign clear ownership for resolution, and establish specific timelines for patient departure. Hospitals that embrace this structured, barrier-focused model can achieve meaningful reductions in length of stay, free up scarce hospital capacity, and improve the experience of patients and staff alike.
